# 1,25-Dihydroxyvitamin D_3_ Inhibits Lipopolysaccharide-Induced Interleukin-6 Production by C2C12 Myotubes

**DOI:** 10.3390/medicina56090450

**Published:** 2020-09-04

**Authors:** Koji Nonaka, Junichi Akiyama, Yoshiyuki Yoshikawa, Satsuki Une, Kenichi Ito

**Affiliations:** 1 Faculty of Health Sciences, Naragakuen University, Nara, Nara 631-8524, Japan; y-yoshi@naragakuen-u.jp (Y.Y.); itok@naragakuen-u.jp (K.I.); 2 Department of Physical Therapy, School of Health Care and Social Welfare, Kibi International University, Takahashi, Okayama 716-8508, Japan; Akiyama@kiui.ac.jp; 3 Faculty of Education, Kagawa University, Takamatsu 760-8521, Japan; une@ed.kagawa-u.ac.jp

**Keywords:** vitamin D, muscle cell, interleukin-6, tumor necrosis factor α, muscle atrophy F-box, muscle ring-finger protein-1, myosin heavy chain

## Abstract

*Background and Objective:* 1,25-dihydroxyvitamin D_3_ (1,25(OH)_2_D_3_) inhibits proinflammatory cytokines in microglial cells and monocytes. However, it is unclear whether 1,25(OH)_2_D_3_ inhibits proinflammatory cytokines in muscle cells. This study was conducted to investigate whether 1,25(OH)_2_D_3_ inhibits the production of proinflammatory cytokines, resulting in inhibition of the protein expression of E3 ubiquitin ligases and muscle protein loss. *Materials and Methods:* C2C12 myoblasts were proliferated in Dulbecco’s modified Eagle medium (DMEM) containing 10% fetal bovine serum, and myoblasts were differentiated into myotubes in DMEM containing 2% horse serum. Myotubes were treated with 1,25(OH)_2_D_3_ for 24 h, followed by lipopolysaccharide (LPS) stimulation for 48 h. *Results:* Interleukin (IL)-6 protein concentrations were higher in the culture supernatant following LPS stimulation compared to that without LPS stimulation (*p* < 0.001). However, the IL-6 concentration was significantly lower in C2C12 myotubes following 1,25(OH)_2_D_3_ treatment than in C2C12 myotubes without 1,25(OH)_2_D_3_ treatment (*p* < 0.001). The myosin heavy chain (MHC), muscle atrophy F-box, and muscle ring-finger protein-1 protein levels did not significantly differ (P = 0.324, 0.552, and 0.352, respectively). We could not compare tumor necrosis factor α (TNFα) protein levels because they were below the limit of detection of our assay in many supernatant samples, including in LPS-stimulated samples. *Conclusions:* 1,25(OH)_2_D_3_ inhibited increases in IL-6 protein concentrations in muscle cells stimulated by LPS, suggesting that 1,25(OH)_2_D_3_ inhibits inflammation in muscle cells. The findings suggest that 1,25(OH)_2_D_3_ can prevent or improve sarcopenia, which is associated with IL-6. The TNFα protein content could not be measured, and MHC was not decreased despite LPS stimulation of C2C12 myotubes. Further studies are needed to examine the effects of higher doses of LPS stimulation on muscle cells and use more sensitive methods for measuring TNFα protein to investigate the preventive effects of 1,25(OH)_2_D_3_ on increased TNFα and muscle proteolysis.

## 1. Introduction

Vitamin D is well known to be involved in maintaining calcium homeostasis and bone metabolism. Vitamin D is supplied to the body via food intake and is synthesized in the skin following exposure to sun rays. In the body, vitamin D is hydroxylated by 25-hydroxylase and converted to 25-hydroxyvitamin D (25(OH)D) in the liver [1]. Second, 25(OH)D is hydroxylated by 1-hydroxlase and converted to 1,25-dihydroxyvitamin D (1,25(OH)_2_D), which is the active form of vitamin D, in the kidney [1]. 1,25(OH)_2_D binds to the vitamin D receptor and induces transcriptional and nongenomic responses [2]. Vitamin D may be also involved in reducing inflammation [3]. In a systematic review and meta-analysis, vitamin D supplementation was described to reduce rheumatoid disease activity [4]. Vitamin D supplementation has been reported as an effective treatment for reducing atopic dermatitis in children [5]. Kabbani et al. [6] performed a five-year follow-up study and observed that low vitamin D levels were associated with inflammatory bowel disease severity. Thus, vitamin D is related to inflammatory diseases. In addition, the effects of vitamin D on inflammation have been studied in vivo and in vitro. The effects of vitamin D on proinflammatory cytokines, such as interleukin-6 (IL-6) and tumor necrosis factor α (TNFα), have been reported in vitro and in vivo. 1,25(OH)_2_D_3_ has been shown to inhibit lipopolysaccharide (LPS)-induced IL-6 mRNA expression in microglial BV-2 cells [7]. 1,25(OH)_2_D_3_ inhibits IL-6 and TNFα protein production in LPS-stimulated human monocytes [8] and reduces the induction of TNFα by LPS/interferon γ in macrophages isolated from C57BL/6 mice [9]. Vitamin D_3_ supplementation has been shown to decrease brain TNFα protein levels in rats with fatty liver [10]. The vitamin D receptor is found in muscle cells [11]. Therefore, 1,25(OH)_2_D_3_ may inhibit the production of proinflammatory cytokines when muscle cells are exposed to inflammatory stimulation; however, this remains unclear.

Loss of muscle protein has been reported to be induced by IL-6 [12,13] and TNFα [14]. Two muscle-specific E3 ubiquitin ligases, muscle atrophy F-box (MAFbx (atrogin-1)) and muscle ring-finger protein-1 (MuRF1), are involved in muscle protein degradation [15]. IL-6 increases MAFbx mRNA levels in the gastrocnemius muscle of mice [16]. Inhibition of IL-6 prevents MuRF1 mRNA expression and ameliorates mouse soleus muscle atrophy induced by tail suspension [17]. TNFα induces MAFbx mRNA expression in C2C12 myotubes [18]. TNFα also increases the mRNA and protein levels of MuRF1 in C2C12 myotubes and the soleus muscle of mice [19]. Taken together, if proinflammatory cytokine production in the muscle is inhibited during inflammatory stimulation, muscle protein loss can be prevented.

In this study, we hypothesized that 1,25(OH)_2_D_3_ inhibits muscle inflammation to prevent muscle protein loss by attenuating E3 ubiquitin ligases. Therefore, this study was conducted to investigate whether 1,25(OH)_2_D_3_ inhibits the production of proinflammatory cytokines, resulting in inhibition of the protein expression of E3 ubiquitin ligases and muscle protein loss. We investigated the effect of 1,25(OH)_2_D_3_ on protein production of IL-6 and TNFα, as proinflammatory cytokines, induced by LPS in C2C12 myotubes, and simultaneously measured the protein levels of myosin heavy chain (MHC), a muscle fibrillar protein, and MAFbx and MuRF1 as E3 ubiquitin ligases.

## 2. Materials and Methods

### 2.1. Cell Culture and Treatment

C2C12 myoblasts (RIKEN Bioresource Center Cell Bank, Tsukuba, Japan) were proliferated in Dulbecco’s modified Eagle medium (DMEM) containing 10% fetal bovine, serum, 1% penicillin, and 1% streptomycin as the growth medium at 37 °C in a 5% CO_2_ air-humidified chamber. As the cells approached confluency, the growth medium was replaced with DMEM containing 2% horse serum, 1% penicillin, and 1% streptomycin as the differentiation medium for four days to differentiate the myotubes. The myotubes were incubated in differentiation medium containing 0, 0.1, 1, or 10 nM of 1,25(OH)_2_D_3_ (031–14281; Wako Pure Chemical Industries, Ltd., Osaka, Japan) dissolved in ethanol at a final concentration of 0.1% in the medium for 24 h. The viability of L6 muscle cells were not affected by 0.5% ethanol exposure for 12 h [20]. Therefore, C2C12 myotubes were likely not affected by 0.1% ethanol exposure for 24 h. Next, the cells were incubated in differentiation medium containing 100 ng/mL LPS (120–05131, Wako Pure Chemical Industries, Osaka, Japan) for 48 h.

### 2.2. IL-6 and TNFα Concentrations

After LPS treatment, the IL-6 and TNFα concentrations in the culture supernatant were measured by enzyme-linked immunosorbent assay (ELISA) using a Mouse IL-6 Assay Kit (#27768; Immuno-Biological Laboratories Co., Ltd., Gunma, Japan) and Mouse TNF-α Instant ELISA (BMS607/2INST; eBioscience, Inc., San Diego, CA, USA), respectively. The measurements were performed according to each of the manufacturers’ instructions. Absorbance was read using a microplate reader (Model 680; Bio-Rad Laboratories, Hercules, CA, USA).

### 2.3. MHC, MuRF1, and MAFbx

The protein expression levels of MHC, MuRF1, and MAFbx were measured by Western blotting. After LPS treatment, the cells were washed three times with phosphate-buffered saline and then lysed and homogenized by ultrasound in 20 mM Tris-HCl buffer, pH 7.6, containing 150 mM KCl, 1% TritonX-100, and cOmplete™ protease inhibitor cocktail (Roche Diagnostics, Basel, Switzerland). The extracts were centrifuged at 12,000× *g* for 10 min at 4 °C, and aliquots of the supernatants were used for Western blot analyses. EzApply (ATTO, Tokyo, Japan) was added to the aliquots, and the samples were boiled for 5 min. The proteins in the samples were separated on a 5–20% gradient polyacrylamide gel (ATTO) by electrophoresis and transferred to polyvinylidene fluoride membranes (ATTO) by the semi-dry blotting method. The membranes were stained with Ponceau-S staining solution (Beacle, Inc., Kyoto, Japan), blocked with EzBlock Chemi (ATTO) for 1 h at room temperature, and incubated with primary antibodies for 1 h at 37 °C. The antibodies used were anti-myosin heavy chain (1:5000; NB300-284; Novus Biologicals, Littleton, CO, USA), anti-MAFbx (1:5000; sc-33782; Santa Cruz Biotechnology, Dallas, TX, USA), and anti-MuRF1 (1:5000; sc-27642; Santa Cruz Biotechnology, Dallas, TX, USA). Following incubation with the primary antibodies, the membranes were washed three times (10 min/wash) in EzWash (ATTO) containing 0.1% Tween 20 and incubated with the secondary antibodies for 1 h at room temperature. The secondary antibodies used were anti-mouse IgG (1:25,000; Nacalai Tesque, Kyoto, Japan) for myosin heavy chain, anti-rabbit IgG (1:25,000; Nacalai Tesque, Kyoto, Japan) for MAFbx, and anti-goat IgG (1:50,000; Abcam, Cambridge, UK) for MuRF1. The membranes were washed three times (10 min/wash) in EzWash containing 0.1% Tween 20 and reacted with ECL Prime Western Blot Detection Reagent (GE Healthcare, Buckinghamshire, UK) for 5 min at room temperature. The protein bands were detected using a LumiCube (Liponics, Inc., Tokyo, Japan) and analyzed using ImageJ software (NIH, Bethesda, MD, USA). The bands from the Ponceau-stained membranes were used as protein loading controls. The data were normalized to the value of the myotubes without treatment with both 1,25(OH)_2_D_3_ and LPS.

### 2.4. Statistical Analysis

All data were expressed as the mean ± standard error of the mean. The data were initially analyzed by one-way analysis of variance (ANOVA), and significant results were further evaluated by Bonferroni post hoc comparison. Statistical analyses were performed using Ekuseru-Toukei 2008 (Social Survey Research Information Co., Ltd., Tokyo, Japan), and *p* < 0.05 was considered as statistically significant.

## 3. Results

### 3.1. IL-6 and TNFα Concentrations

**Figure 1:**
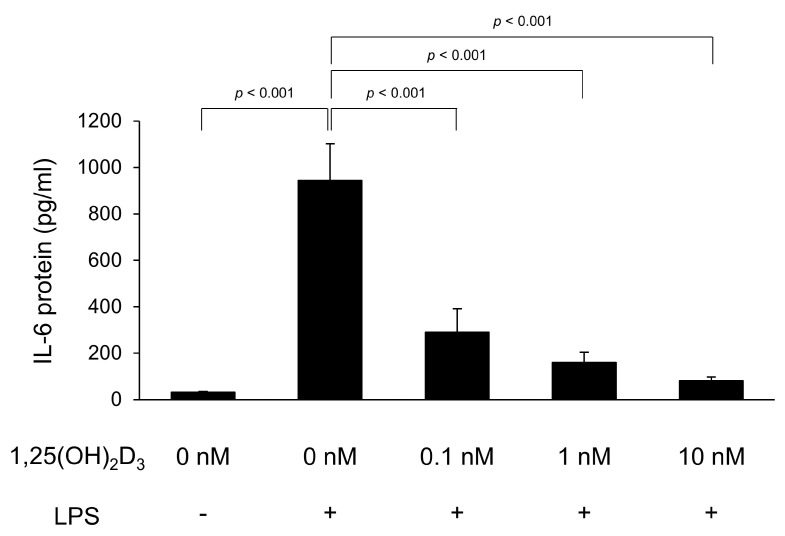
1,25-Dihydroxyvitamin D_3_ (1,25(OH)_2_D_3_) inhibits lipopolysaccharide (LPS)-induced interleukin-6 (IL-6) production in C2C12 myotubes. C2C12 myotubes were cultured with 1,25(OH)_2_D_3_ for 24 h and then stimulated by LPS for 48 h. IL-6 protein levels in the culture supernatants following LPS stimulation were measured by enzyme-linked immunosorbent assay (*n* = 7). Values are expressed as the mean ± standard error of the mean.

To verify the protein production of proinflammatory cytokines, IL-6 and TNFα protein concentrations in the culture supernatant of C2C12 myotubes were measured by ELISA. The IL-6 protein concentration is shown in Figure 1. LPS significantly increased IL-6 protein levels (944 ± 158 pg/mL) in the culture supernatant compared to in the culture supernatant without LPS stimulation (31 ± 2 pg/mL, *p* < 0.001). However, the IL-6 concentration was significantly low in C2C12 myotubes with 0.1, 1, and 10 ng/mg of 1,25(OH)_2_D_3_ treatment (IL-6 concentration; 289 ± 101, 160 ± 45, and 81 ± 17 pg/mL, respectively) compared to that in untreated C2C12 myotubes (*p* < 0.001). Unfortunately, we did not compare the TNFα protein levels because they were below the limit of detection of our assay (31.3 pg/mL) in many supernatant samples.

### 3.2. MHC, MuRF1, and MAFbx

**Figure 2:**
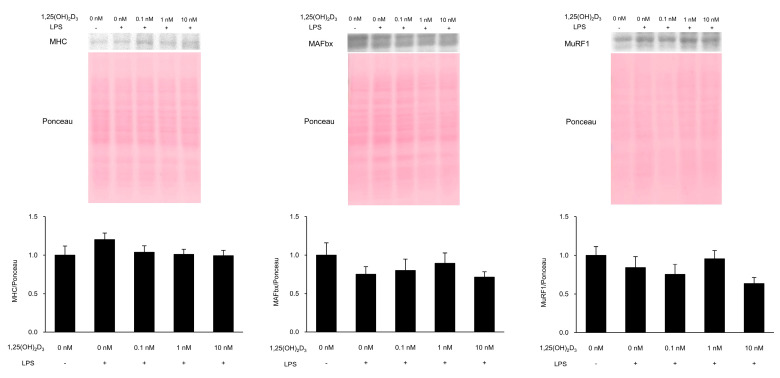
Myosin heavy chain (MHC), muscle atrophy F-box (MAFbx), and muscle ring-finger protein-1 (MuRF1) protein levels. C2C12 myotubes were cultured with 1,25(OH)_2_D_3_ for 24 h and then stimulated by lipopolysaccharide (LPS) for 48 h. Protein levels of MHC, MAFbx, and MuRF1 in the cells following LPS stimulation were measured by Western blotting (n = 5–6). Values are expressed as the mean ± standard error of the mean.

To verify muscle proteolysis and E3 ubiquitin ligase levels, we measured the loss of MHC as a muscle protein and MAFbx and MurF1 as E3 ubiquitin ligases by Western blotting (Figure 2). MHC, MAFbx, and MuRF1 protein levels were not significantly different according to one-way ANOVA (*p* = 0.324, 0.552, and 0.352, respectively).

## 4. Discussion

This study was performed to investigate whether 1,25(OH)_2_D_3_ inhibits muscle cell inflammation to result in muscle protein loss. The findings of this study were as follows: (i) 1,25(OH)_2_D_3_ inhibited IL-6 production induced by LPS in C2C12 myotubes; (ii) TNFα protein levels were low in many C2C12 myotube samples; and (iii) LPS did not cause MHC loss and failed to increase MAFbx and MuRF1 protein levels in C2C12 myotubes. These findings suggest that 1,25(OH)_2_D_3_ has an anti-inflammatory effect on muscle cells. However, it is unclear whether 1,25(OH)_2_D_3_ inhibits muscle protein loss induced by inflammatory stimulation.

IL-6 protein levels were increased in the supernatant following LPS stimulation of C2C12 myotubes. Our results were similar to those of studies in which LPS stimulation of C2C12 cells increased IL-6 protein production [21]. Interestingly, 1,25(OH)_2_D_3_ inhibited IL-6 production when C2C12 myotubes were treated with LPS. 1,25(OH)_2_D_3_ has been reported to inhibit increases in IL-6 production when human monocytes were treated with LPS [8]. Our results suggest that muscle cells treated with 1,25(OH)_2_D_3_ also showed inhibitory effects on IL-6 protein production following exposure to LPS. Therefore, 1,25(OH)_2_D_3_ may also play roles in preventing inflammation in muscle cells.

TNFα protein could not be measured because of its low levels in the culture supernatant samples. Baker et al. [21] reported that TNFα protein levels increased when C2C12 myotubes were stimulated with LPS. Frost et al. [22] reported that the protein level of TNFα in extracts and the supernatant of C2C12 cells with or without LPS stimulation was below the detection limit of their assay. IL-6 infusion did not increase endotoxin-induced TNFα production in humans [23], indicating that IL-6 inhibits TNFα protein production induced by LPS stimulation. The reasons why TNFα protein could not be measured in many samples, including those stimulated by LPS, were considered to be as follows: (i) TNFα protein production in muscle cells may be relatively low, (ii) TNFα protein was not markedly increased by LPS stimulation of C2C12 myotubes, and/or (iii) increased IL-6 protein by LPS stimulation inhibited TNFα protein production. It remains unclear whether 1,25(OH)_2_D_3_ inhibits TNFα protein levels in muscle cells stimulated by LPS.

MHC protein levels were not decreased by LPS stimulation in C2C12 cells. This indicates that muscle protein loss did not occur following LPS stimulation. Doyle et al. [24] reported a decrease in MHC protein levels by LPS stimulation of C2C12 myotubes, which contradicts our results. Muscle proteolysis is associated with the E3 ubiquitin ligases MAFbx and MuRF1 [15]. MAFbx and MuRF1 mRNA levels were not significantly different between rat skeletal muscle with and without infusion of IL-6 [13]. Although MAFbx mRNA was increased by 100 ng/mL of IL-6 and MuRF1 mRNA was significantly increased by 10 and 100 ng/mL IL-6, MAFbx mRNA was not increased by 1 or 10 ng/mL of IL-6 and MuRF1 mRNA was not increased by 1 ng/mL of IL-6 in human myocytes [25]. In this study, LPS increased IL-6 levels by up to approximately 0.94 ng/mL, which may be too low to increase MAFbx and MuRF1 protein levels. TNFα has been shown to increase MAFbx and MuRF1 mRNA expression in C2C12 myotubes [26]. Taken together, both IL-6 and TNFα may not have activated E3 ubiquitin ligases, and thus, muscle proteolysis did not occur in the present study. Therefore, whether 1,25(OH)_2_D_3_ prevents muscle protein loss by inhibiting proinflammatory cytokine-induced increases in E3 ubiquitin ligases when muscle cells are stimulated with LPS remains unclear.

The limitations of this study were that the TNFα protein content could not be measured, and MHC was not decreased despite LPS stimulation of C2C12 myotubes. This likely occurred because the method used to measure TNFα had low sensitivity and because the LPS concentration was too low to induce proteolysis. Additional studies are needed to examine the effects of higher doses of LPS stimulation on muscle cells and use more sensitive measurement methods for detecting TNFα protein to investigate the preventive effects of 1,25(OH)_2_D_3_ on increased TNFα and muscle proteolysis.

## 5. Conclusions

In this study, we investigated whether 1,25(OH)_2_D_3_ has anti-inflammatory effects in muscle cells and prevents proteolysis induced by inflammatory stimulation. 1,25(OH)_2_D_3_ inhibited increases in IL-6 protein levels in muscle cells when stimulated by LPS, suggesting that 1,25(OH)_2_D_3_ inhibits inflammation in muscle cells. IL-6 is associated with sarcopenia [27]. Therefore, our findings suggest that 1,25(OH)_2_D_3_ can prevent or improve sarcopenia by inhibiting IL-6 production. However, in this study, the effects of 1,25(OH)_2_D_3_ on TNFα and muscle protein loss in muscle cells remain unclear, warranting further studies.
